# Molecular profile of dissociative drug ketamine in relation to its rapid antidepressant action

**DOI:** 10.1186/s12864-016-2713-3

**Published:** 2016-05-17

**Authors:** Joanna Ficek, Magdalena Zygmunt, Marcin Piechota, Dzesika Hoinkis, Jan Rodriguez Parkitna, Ryszard Przewlocki, Michal Korostynski

**Affiliations:** Department of Molecular Neuropharmacology, Institute of Pharmacology PAS, Smetna 12, Krakow, 31-343 Poland

**Keywords:** NMDA antagonists, Gene expression profile, Striatum, Hippocampus

## Abstract

**Background:**

The NMDA receptor antagonist ketamine was found to act as a fast-acting antidepressant. The effects of single treatment were reported to persist for days to weeks, even in otherwise treatment-refractory cases. Identification of the mechanisms underlying ketamine’s antidepressant action may permit development of novel drugs, with similar clinical properties but lacking psychotomimetic, sedative and other side effects.

**Methods:**

We applied whole-genome microarray profiling to analyze detailed time-course (1, 2, 4 and 8 h) of transcriptome alterations in the striatum and hippocampus following acute administration of ketamine, memantine and phencyclidine in C57BL/6 J mice. The transcriptional effects of ketamine were further analyzed using next-generation sequencing and quantitative PCR. Gene expression alterations induced by the NMDA antagonists were compared to the molecular profiles of psychotropic drugs: antidepressants, antipsychotics, anxiolytics, psychostimulants and opioids.

**Results:**

We identified 52 transcripts (e.g. *Dusp1*, *Per1* and *Fkbp5*) with altered expression (FDR < 1 %) in response to treatment with NMDA receptor antagonists. Functional links that connect expression of the regulated genes to the MAPK, IL-6 and insulin signaling pathways were indicated. Moreover, ketamine-regulated expression of specific gene isoforms was detected (e.g. *Tsc22d3*, *Sgk1* and *Hif3a*). The comparison with other psychotropic drugs revealed that the molecular effects of ketamine are most similar to memantine and phencyclidine. Clustering based on expression profiles placed the NMDA antagonists among fluoxetine, tianeptine, as well as opioids and ethanol.

**Conclusions:**

The identified patterns of gene expression alteration in the brain provided novel molecular classification of ketamine. The transcriptional profile of ketamine reflects its multi-target pharmacological nature. The results reveal similarities between the effects of ketamine and monoaminergic antidepressants that may explain the mechanisms of its rapid antidepressant action.

**Electronic supplementary material:**

The online version of this article (doi:10.1186/s12864-016-2713-3) contains supplementary material, which is available to authorized users.

## Background

Ketamine is a dissociative anaesthetic that causes profound analgesia and amnesia. Recently, there has been growing interest in the reported antidepressant effects of ketamine. Due to the almost immediate onset of antidepressant effects ketamine could potentially be particularly useful in acute treatment of depression [1]. This rapid-onset antidepressant action provides a novel potential approach for treatment-resistant major depressive disorder (MDD). However, clinical application of ketamine is limited by its psychotomimetic effects and rewarding properties, which are responsible for its recreational use and abuse [2].

Ketamine is a noncompetitive antagonist of the NMDA glutamate receptor, with affinities for other receptors and transporters, including dopamine D2 and opioid receptors as well as monoamine transporters [3–6]. The complex pharmacological profile of ketamine makes it difficult to postulate a specific mechanism of its rapid antidepressant effect. The comparison with other NMDA receptor antagonists does not provide clear indication of the possible mechanism of action [7, 8]. Phencyclidine (PCP) induces cognitive disruption and psychotic-spectrum reactions but, unlike ketamine, was not observed to have antidepressant effects in humans. Memantine, a drug with moderate-affinity for NMDA receptors and without the adverse effects, also does not show evidence of rapid antidepressant effects in depressed patients [9]. Thus, the antidepressant effects are specific to ketamine and result from unique combination of its receptor targets and triggered neuronal mechanisms. Elucidating the mechanism of ketamine action would provide an important insight into the molecular correlates underlying the efficacy of rapid antidepressants and also suggest a useful approach for screening of novel compounds.

Ketamine facilitates neuronal plasticity in brain areas implicated in MDD, including the hippocampus, the prefrontal cortex and the striatum. It has been shown that ketamine enhances mammalian target of rapamycin (mTOR) and increases expression of the brain-derived neurotrophic factor (BDNF), resulting in modification to the number and function of synaptic connections [10, 11]. These alterations are related to activation of downstream intracellular signaling pathways and transcription of genes [12]. Therefore, the analysis of transcriptional programs associated with rapid action of ketamine may provide novel insight into the molecular mechanisms underlying specific behavioral effects of this drug.

In this study, we used gene expression profiling to compare ketamine with PCP and memantine based on their transcriptional effects in the striatum and hippocampus. Furthermore, we have analyzed the molecular profile of the selected NMDA antagonists in comparison to over a dozen of antidepressants, antipsychotics, psychostimulants, anxiolytics and opioids [13]. Our results identify differences as well as similarities between gene expression alterations induced by ketamine and other psychotropic drugs that may explain the unique psychoactive properties of ketamine and its rapid antidepressant effects. This study provides also a molecular classification of ketamine among psychotropic drugs based on the whole-transcriptome expression profile.

## Results

### Time-course of gene expression alterations induced by the selected NMDA receptor ligands

**Fig. 1:**
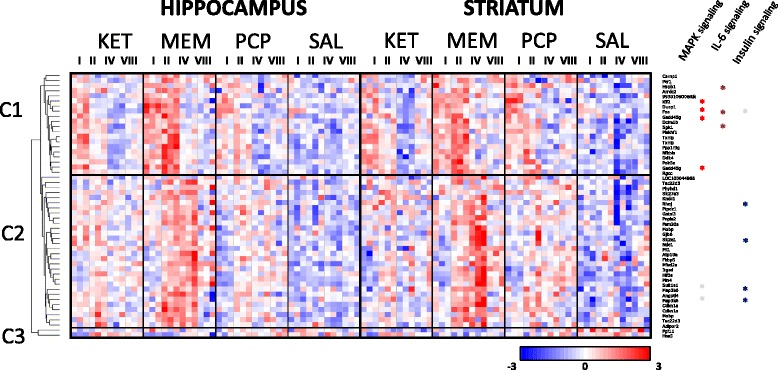
Gene expression alterations induced by NMDA receptor antagonists in mouse hippocampus and striatum. Microarray results are shown as a heat map and include genes with a genome-wide significance from three-way ANOVA for the drug factor (FDR < 0.01 and log_2_ fold change > 0.5 at least at one time-point in any substance). Colored rectangles represent transcript abundance 1, 2, 4 and 8 h after injection of the drug indicated above - ketamine (KET), memantine (MEM) and PCP. Intensity of the color is proportional to the standardized values (between −3 and 3) from each microarray, as displayed on the bar below the heat map image. Hierarchical clustering was performed using Euclidean distance. Major clusters of drug-responsive genes are arbitrarily described as ‘C1’, ‘C2’ and ‘C3’. The over-representation of the pathways was found statistically significant with *p < 0.05. Genes involved in the indicated pathways, but displaying different transcription patterns were marked with grey color

First, we have analyzed alterations in the gene expression profile in the striatum and hippocampus following single administration of ketamine, memantine or PCP. The analysis of alterations in mRNA abundance levels was performed using whole-genome Illumina WG-6 microarrays at four time points (1, 2, 4 and 8 h following drug injection). Array data were subjected to three-way analysis of variance (ANOVA) with drug*,* time and tissue as factors (Additional file 1). We found 52 drug-responsive transcripts (Fig. 1) at the threshold of 1 % FDR (nominal *p* = 1.15 × 10^−5^) with fold change greater than 0.5 (at least at one time point for one of the drugs). We found moderately higher transcriptional effects of memantine treatment comparing to ketamine and PCP (Additional file 2). At the same statistical threshold of 1 % FDR, 258 transcripts with differential expression for the time factor (nominal *p* = 5.6 × 10^−5^) and 21.316 transcripts for the tissue factor (nominal *p* = 6.4 × 10^−3^) were identified. The results for the time factor (4 time points) indicated genes altered during the diurnal cycle such as e.g. *Dbp*, *Ciart* and *Per2*. The observed distinction between basal gene expression profiles in the striatum and the hippocampus was in line with expectations. The largest number of differences in gene expression was observed in case of neuron-type specific markers e.g. *Pdyn*, *Rgs9* and *Tac1*.

### Hierarchical clustering and functional enrichment analysis of drug-responsive genes

Hierarchical clustering revealed three clusters of drug-regulated genes (arbitrarily labeled as C1, C2 and C3). Gene clusters C1 and C2 consisted of transcripts with increased mRNA abundance levels in response to drug treatment (Fig. 1). Cluster C1 was induced 1 h after injection of ketamine and PCP and 1 to 2 h after injection of memantine. Cluster C2 was induced 2 to 4 h by the three selected drugs to different degrees. Example genes from each cluster include: *Dusp1*, *Per1*, *Sgk1* (C1) and *Fkbp5*, *Slc2a1*, *Map3k6* (C2). Cluster C3 includes two down-regulated genes *Fgf11* and *Hes5*. In addition, functional enrichment analysis was used to investigate cell signaling pathways related to drug-induced gene expression patterns (as shown in Fig. 1, right side). Enrichr, a gene signature search tool based on the WikiPathways database, indicated overrepresentation of genes involved in MAPK (*p* = 0.0005) and IL-6 (*p* = 0.002) signaling pathways among C1 gene cluster. Furthermore, the enrichment analysis found genes from C2 cluster to be involved in insulin signaling (*p* = 0.004; Additional file 3).

### The comparison of transcriptional effects in the striatum and hippocampus

The overall differences in drug-induced gene expression between striatum and hippocampus were compared using two-way ANOVA for drug and time factors performed separately in each tissue. We found 62 transcripts regulated by drug treatment in the hippocampus and 62 transcripts altered in the striatum (at *p* < 0.0005, absolute fold > 0.3 at least at one time point). The examples of regulated genes are *Dbp* and *Mat2a* in the hippocampus, and *Fgf11* and *Scrt2* in the striatum. We found 19 of the genes to be regulated in both the analyzed tissues, including *Dusp1*, *Sgk1* and *Sult1a1* (Additional file 4). The pattern of gene expression alterations over time was similar for all the selected NMDA antagonists (Additional file 5). In the hippocampus the most substantial increase in mRNA abundance levels was observed 1 h following the administration of ketamine, 2 h after memantine and 2 to 4 h after PCP. The strongest response to each drug in the striatum was observed at 2 to 4 h after injection. Time course of expression alterations of down-regulated genes corresponded to the pattern observed for up-regulated genes.

### The comparison of transcriptional effects of the selected NMDA antagonists

**Fig. 2:**
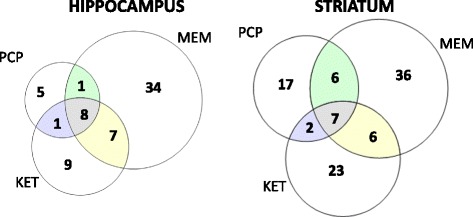
Comparison of the number of genes regulated by ketamine, memantine and PCP in the hippocampus and striatum. Venn diagrams show the number and overlap of genes altered by drug treatment in the mouse hippocampus and striatum. Lists of transcripts with statistical significance *p* < 0.0005 for drug factor after two-way ANOVA in each tissue and absolute fold change greater than 0.3 over saline control (at one of the time points) were analyzed (Additional file 2)

To compare differences and similarities in gene expression after administration of ketamine, memantine and PCP we performed two-way ANOVA with drug and time factors for each drug and tissue separately (Fig. 2). The similarities in molecular effects of the selected NMDA antagonists are presented on Venn diagrams. The overlap between each pair of drugs was tested using Fisher’s exact test and proved to be significant in each case (*p* < 0.01). In the hippocampus, the analysis indicated 25, 50 and 15 drug-responsive transcripts after treatment with ketamine, memantine and PCP respectively (at *p* < 0.0005, absolute fold > 0.3 at least at one time point). We found 8 genes to be regulated by all of these compounds, including *Plin4*, *Ppp1r3g* and *Cdkn1a*. In the striatum 38 transcripts were differentially expressed after administration of ketamine, 55 after memantine and 32 after PCP at the same statistical threshold (at *p* < 0.0005, absolute fold > 0.3). The analysis indicated 7 genes altered by all the selected drugs in the striatum, for instance *Sgk1*, *Fgf11* and *Dnah2* (Additional file 2).

### Gene expression profile of ketamine in comparison to major classes of psychotropic drugs

**Fig. 3:**
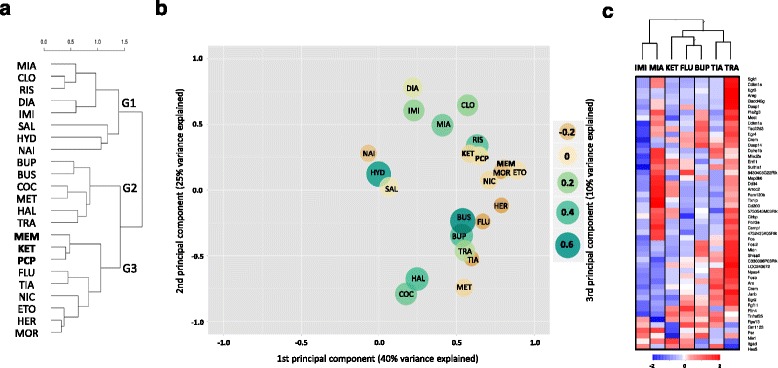
Comparison of ketamine, memantine and PCP to other psychotropic drugs based on gene expression patterns in the mouse striatum. Cluster dendrogram (**a**), PCA plot (**b**) and heat map (**c**) were generated based on expression alterations of the top 50 drug-responsive genes found statistically most significant. The gene expression profiles of ketamine (KET), memantine (MEM) and PCP (PCP) were combined with previously published profiles of 18 psychotropic drugs from diverse pharmacological and therapeutic classes: mianserin (MIA), imipramine (IMI), fluoxetine (FLU), bupropion (BUP), tianeptine (TIA), tranylcypromine (TRA), methamphetamine (MET), cocaine (COC), nicotine (NIC), heroin (HER), morphine (MOR), ethanol (ETO), diazepam (DIA), buspirone (BUS), hydroxizine (HYD), clozapine (CLO), risperidone (RIS) and haloperidol (HAL), with saline (SAL) and naive (NAI) groups as the controls [13]. The genes were selected based on ANOVA results for drug factor (top 50) at a threshold of nominal *p* < 10^−26^ (after Bonferroni correction *p* < 10^−21^). Hierarchical clustering (**a**) was performed using the measure of Pearson correlation distance and complete distance linkage methods. The PCA plot (**b**) displays the pattern of similarity between the selected drugs. The first and second components are shown on x and y axis respectively, while the values of the third component are coded by color and size of the circles as indicated on the legend. A comparison of ketamine to antidepressants (**c**) was based on the patterns of drug-induced transcriptional alterations in the striatum (at 2 h after the treatment). Drug effects were balanced using modified minimum-maximum normalization, where the maximum was set up as average from the top 10 fold changes for every drug. Colored rectangles represent expression levels of the top 50 drug-regulated genes indicated on the right

We performed a comparative analysis of ketamine, memantine and PCP induced gene expression with data from our previous study, which examined the expression profiles of 18 drugs including antidepressants, antipsychotics and drugs of abuse [13]. Top 50 genes were identified using two-way ANOVA for drug and time factors on combined data followed by correction for multiple testing (corrected *p* < 2.3 × 10^−21^) and then the obtained results were further used for comparison of the selected psychotropic drugs. Expression pattern comparison was performed among sets of the top 50 drug-regulated genes (using data for all the 21 drugs). Hierarchical clustering and principal component analysis (PCA) were performed to classify ketamine among other psychotropic drugs based on their gene expression profiles (Fig. 3). The clustering indicated three major groups (G1-G3) that contained clinically and pharmacologically divergent drugs, including (G1) anxiolytics, atypical antipsychotics, imipramine and mianserin; (G2) psychostimulants, tranylcypromine, bupropion, haloperidol and buspirone; (G3) NMDA antagonists, fluoxetine, tianeptine as well as nicotine, ethanol, heroin and morphine (Fig. 3a). The gene expression profile of ketamine was most similar to those of memantine and PCP. The three examined compounds acting at NMDA receptors were clustered with two antidepressants and the drugs of abuse.

Three main components of PCA explained 58 % of variance in gene expression (Fig. 3b). In terms of all three components, the gene expression signature of ketamine was found to be most similar to PCP, memantine, opioids, ethanol and nicotine. Some similarities in gene expression profiles were found between ketamine and risperidone (1st and 2nd components) as well as ketamine, fluoxetine and tianeptine (3rd component). Based on the lists of genes correlated with the PCA components, we assume that the first component represents drug-induced expression of GR-dependent genes (explaining 30 % of variance), while the second component describes the pattern of expression of activity-dependent genes (explaining 19 % of variance). The third component was identified to reflect prolonged drug-dependent induction of activity-dependent genes (2 and 4 h after treatment).

In addition, we have directly compared molecular profile of ketamine with six antidepressant drugs with different pharmacological profiles (imipramine, mianserin, fluoxetine, bupropion, tianeptine and tranylcypromine, Fig. 3c). The analysis was performed using expression data from the peak (2 h after the drug administration of transcriptional effects of ketamine) which corresponds with the onset of antidepressant effects in humans [14]. In general, antidepressant drugs induced diversified profiles of gene expression alterations in the striatum. Gene expression induced by ketamine was most similar to antidepressants blocking monoamine reuptake by inhibition of SERT and DAT, respectively fluoxetine and bupropion. On the other hand ketamine profile was most distant from imipramine and mianserin.

### Search for ketamine-regulated expression of specific transcriptional variants

In order to comprehensively examine ketamine-induced gene expression at the level of separate transcriptional units we used next-generation sequencing. From the list of 52 transcripts identified by microarray profiling, 23 genes regulated by ketamine (three-way ANOVA *p* < 0.05) at 2 h after treatment (absolute fold over saline control > 0.2) were selected. The mRNA abundance levels of 79 transcripts assigned to 23 genes were extracted from the whole-transcriptome dataset. Significant differences in gene expression between ketamine- and saline-treated controls (*p* < 0.05) were identified for 15 genes. Six of these (*Plin4*, *Nfkbia*, *Plekhf1*, *Rgcc*, *Csrnp1* and *Arrdc2*) express a single form of mRNA contained constitutive exons (CNE). Nine genes showed ketamine-dependent regulation of alternative transcriptional forms. Classifications of transcription indicated different types of alternative expression of the regulated genes. For *Sgk1*, *Tsc22d3* and *Hif3a* expression of variants with alternative transcriptional start sites and first exons (AFE) was detected. The ketamine-induced expression of specific isoform with alternative last exon (ALE) and termination site was found for *Rhoj*. Several genes express variants with retained intron (IR), including *Slc2a1*, *Gjb6* and *Map3k6*. Moreover, an analysis on the exon level indicated regulation of *Slc2a1* variants with mutually exclusive exons (MXE). Ketamine-induced regulation of the expression of selected genes on the level of genes, transcriptional units and exons is presented in supplementary results (Additional file 6).

### Validation of ketamine-induced alterations in gene expression

**Fig. 4:**
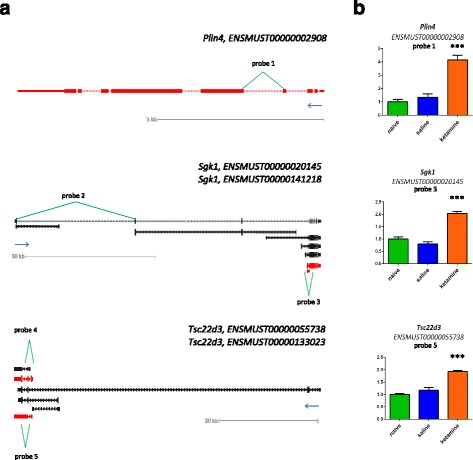
Validation of ketamine-induced gene expression alterations. The expression of *Plin4*, *Sgk1* and *Tsc22d3* in response to ketamine was measured in the mouse striatum. **a** Ketamine-induced expression of specific transcriptional variants was analyzed using next-generation sequencing (*n* = 4). Variant classification indicated different biotypes of regulated transcripts (depicted in red) (Additional file 6). **b** The changes in mRNA abundance levels were measured 2 h after the administration of ketamine, saline or in naive animals. qPCR analyses were performed using samples from an independent biological experiment (*n* = 8). TaqMan probes allow to distinguish between specific transcriptional variants: qPCR probe “1” spans an exon junction of exons 4 and 5 of *Plin4* (ENSMUST00000002908) transcript, qPCR probe “2” spans exons 1 and 2 in *Sgk1* (ENSMUST00000120509), qPCR probe “3” spans exons 1 and 2 in *Sgk1* (ENSMUST00000020145), qPCR probe “4” span exons 1 and 2 in *Tsc22d3* (ENSMUST00000112995), qPCR probe “5” spans exons 1 and 2 in *Tsc22d3* (ENSMUST00000055738). Bars indicate S.E.M, *** *p* < 0.001, one-way ANOVA drug factor

We used quantitative real-time reverse transcription polymerase chain reaction (qPCR) to validate the results obtained using high-throughput methods. The experiments were performed using aliquots of the non-pooled total RNA (*n* = 6) as well as independent biological replicates (*n* = 8). The microarray analysis of ketamine effects in the brain indicated 12 transcripts regulated at the threshold FDR < 1 %. From this list, three top novel ketamine responsive genes *Plin4*, *Sgk1* and *Tsc22d3* were selected for validation (Fig. 4). The changes in mRNA abundance levels were measured in the striatum 2 h after ketamine or saline administration and in naive animals. The treatment factor in ANOVA was significant for all of the transcripts examined by qPCR (Fig. 4b). The effects of ketamine were similar in two independent experiments. Greater than 3-fold induction of perilipin 4 (*Plin4*) mRNA after the treatment was confirmed. Equal upregulation (about 1.5- to 2-fold) of transcripts for serum/glucocorticoid regulated kinase 1 (*Sgk1*) and TSC22 domain family protein 3 (*Tsc22d3*) was observed after acute ketamine administration in the striatum. Moreover, regulation of the specific variants with alternative first exons (AFE) for *Sgk1* and *Tsc22d3* was validated using qPCR (Fig. 4a).

## Discussion

Clinical trials showed that ketamine can induce an antidepressant response within hours from administration [1]. The antidepressant effects may persist up to two weeks and has been effective in treatment-resistant patients [14]. These observations provide hope for potential use of NMDA receptor antagonists in affective disorders. Ketamine has also been effective in treatment of multiple severe neuropsychiatric conditions including bipolar disorder, post-traumatic stress disorder and chronic pain [15, 16]. However, the psychotomimetic and addictive properties of this drug limit its clinical applications [17]. The neurobiological mechanisms related to various clinical actions of this prototypic glutamatergic “rapid antidepressant” are elusive. A major difficulty in identifying the underlying mechanism of ketamine action arises from its complex pharmacological profile [5]. A dissection of ketamine actions on the molecular level may provide insight into particular mechanisms underlying different behavioral effects. Despite the common use of ketamine in clinical and experimental studies there are relatively few reports of its action on gene expression in brain regions involved in the control of motivation and executive functions [18, 19].

Here, we present the results of ketamine-induced gene expression profiling and a comparison with psychotropic drugs from all the major clinical and pharmacological classes. Additionally, the molecular profile of ketamine was analyzed on the level of alternative transcriptional variants using whole-transcriptome resequencing technology. This study was designed to analyze time course of gene expression changes in response to ketamine, PCP and memantine in the striatum and hippocampus. We found that the three factors (drug, time and tissue) contributed to different extents to the profile of gene transcription in the brain. Most differences were observed between the analyzed tissues, however extensive alterations were also related to the diurnal cycle. These results are in agreement with previous studies describing that in the brain, the tissue factor is the main source of variance in expression profiling experiments [20]. Nevertheless, we found that the effects of the selected NMDA antagonists are similar in both the analyzed regions, striatum and hippocampus, in terms of top regulated genes. Both these regions receive dopaminergic inputs from the ventral midbrain that may explain similar transcriptional alterations in response to the selected psychoactive compounds [21]. The time course of gene expression alterations was similar for all the NMDA antagonists with maximum response observed at 2 to 4 h after treatment. The changes of mRNA abundance induced by the NMDA antagonists subsided within 8 h following drug administration. This is consistent with our previous observation that drug-induced gene expression is transient [13]. Moreover, we also found that profiles of gene transcription alterations induced by ketamine, PCP and memantine are similar. This observation may indicate that the main effects of the tested drugs on gene expression are mediated by the blockade of the NMDA receptor.

Previously published microarray results has been heterogeneous in terms of time and tissue selection. A comparative meta-analysis is, therefore, difficult to perform due to different experimental designs and microarray platforms used. For example, gene microarrays were used to assess the effects of PCP and methamphetamine treatment in cerebral cortices. The authors found that these two compounds regulate a list of functionally heterogeneous genes [22]. The identified alterations in gene expression were linked to psychotomimetic potential of PCP and methamphetamine. Other studies analyzed the neurotoxic effects of ketamine and PCP. The effects of these two NMDA receptor antagonists on gene expression were analyzed in the developing rat brain in the context of schizophrenia-like symptoms in animal model [18]. Gene expression profiling in rat limbic cortex of uncompetitive NMDA antagonists (memantine and MK-801) indicated broad actions of these agents on the molecular level [19]. The alterations in mRNA abundance levels were also analyzed to reveal molecular mechanisms of neuroprotective effects of memantine. Several gene expression profiling studies of NMDA antagonists’ effects were performed after repeated administration. The expression of selected genes was analyzed after acute and repeated treatment in the cortex, striatum and hippocampus. It was found that chronic ketamine treatment regulates GABAA receptor alpha 5 subunits in the mouse prefrontal cortex [23].

Interestingly, among the 52 differentially regulated transcripts were genes encoding proteins associated with intracellular pathways associated with the development of mood disorders, MAPK and IL-6 signaling. The first group includes, for instance, the transcript of the dual-specificity phosphatase-1 (DUSP1), which is a negative regulator of MAPK kinase signaling - one of the major pathways that mediate neuronal plasticity [24]. The second group includes the transcript of the serum- and glucocorticoid-inducible kinase 1 (SGK1), which is implicated in memory formation as well as function of glial cells [25, 26]. Both these pathways were previously proposed as factors involved in the mechanisms of depressive disorders associated with chronic stress [27–29]. Other group of transcripts regulated in response to NMDA antagonists contains genes involved in insulin-mediated cellular signaling pathway, including glucose transporter *Slc2a1*. The regulation of this pathway is potentially interesting in the context of connection between depression and insulin resistance in diabetic patients [30].

The observed effects on gene expression may correspond to alterations detected on the protein level. Previous results suggested involvement of molecular mechanisms concerning mTOR, BDNF and eEF2 kinase pathways in ketamine action [11, 31]. These processes are related to the development of depression through synaptic remodeling and immunomodulation [32]. Ketamine has been shown to induce expression of synapse-associated proteins through the rapid activation of mTOR-regulated ERK and Akt signaling pathways [11]. Moreover, mTOR signaling is involved in the resetting of the circadian clock by regulating the expression of circadian proteins [33]. The elevated transcription of the period 1 (*Per1*) gene in response to ketamine may be a consequence of increased activity of mTOR-pathway. Both the molecular levels of regulation, transcripts and proteins expression, form a control system for drug-induced plastic alterations in the brain. In summary, our results show that ketamine affects expression of several genes potentially involved in neuronal plasticity. It should be noted though, that these changes are not exclusive to ketamine and are also observed in case of other NMDA antagonists we tested.

There were several cases where treatment with NMDA antagonists affected the expression of GR-dependent transcripts that are also associated with development of neuropsychiatric disorders. The *Fkbp5* gene is a prime example, as the gene was connected with sensitivity to rapid effects of treatment with antidepressants [34]. Expression of specific Fkbp5 variants is GR-dependent while at the same time the FKBP5 protein is a critical regulator of GR activity [35]. Further examples include the hypoxia inducible factor 3 alpha subunit (*Hif3a*), whose expression was found to be increased in schizophrenic and bipolar patients [36] or *Sgk1* and *Tsc22d3*, which were associated with high interleukin (IL)-6 levels in patients suffering from the major depressive disorder [37]. In all three cases ketamine treatment induced only expression of the short transcriptional variants, which again points at the importance of analysis of gene expression at transcript rather than gene level [38]. The results indicate that ketamine may activate the hypothalamic-pituitary-adrenal axis and release of glucocorticoids from adrenal glands [25]. Thorough analysis of this systemic response may provide further insight into the interaction between the effects of psychotropic drugs, stress and glucocorticoids. The observed gene expression alterations reveals several aspects of the complex molecular profile of drug-induced alterations in the brain.

Despite the similarity between the NMDA antagonists, we identified transcripts with diverse regulation induced by these drugs in the selected brain regions. Differences between ketamine, memantine and PCP may provide important information for the understanding of rapid antidepressant effects. PCP decreases immobility time, a depression-like behavior in the forced swim test in mice, but elicits more intense psychotomimetic effects than ketamine. On the other hand, memantine did not show fast antidepressant-like properties in animal models [10]. Our findings have raised concerns regarding the possibility to dissect unique pattern of ketamine effects by using the comparison to other psychotropic drugs. We and others used gene expression profiling to predict properties of the psychoactive compounds [13, 39]. Here, we used the large microarray data-set to compare ketamine effects with 20 psychotropic drugs from various clinical and pharmacological groups.

The profiles of gene expression alterations induced by the tested NMDA antagonists were most similar to some antidepressants and drugs of abuse. Specifically, the profiles were similar to the monoaminergic antidepressants, fluoxetine and tianeptine. The anti-anhedonic and antidepressant effects of ketamine are rapid in comparison to typical antidepressants. However, the similarities in the patterns of drug-induced gene expression may indicate shared neurobiological effects. A recent clinical report indicated that response to antidepressant treatment is accelerated by the co-administration with methylphenidate [40]. It is possible that the combination of various ketamine effects, including boosting serotonin and dopamine activities, may lead to earlier manifestation of antidepressant action [5, 41]. As it was anticipated, the NMDA antagonists reveal relatively distinct molecular profiles from neuroleptics and atypical antidepressant mianserine. Interestingly, the gene expression profile of ketamine was more similar of opioids, ethanol and nicotine than to psychostimulants. It was previously described that ketamine has mild agonistic activity on opioid receptors and modifies the responsiveness [42]. These effects of ketamine on opioid system may result in expression of genes also regulated in the striatum by opioids and ethanol [43]. Thus, this observation supports the claim that ketamine may have addictive potential which substantially limits its clinical application.

## Conclusions

Gene profiling in limbic brain areas indicated several novel aspects of molecular action of ketamine as well as two other tested NMDA antagonists, memantine and PCP. The presented molecular classification of these drugs positioned them between antidepressants and drugs of abuse. We believe that the obtained profile of ketamine-induced gene expression reflects combination of its pharmacological and neurobiological properties. The unique clinical profile of ketamine that includes psychotomimetic, anti-anhedonic and addictive effects is associated with specific transcriptional alterations. This combination of pharmacological and molecular effects might be difficult to achieve by other compounds even with similar chemical characteristics.

## Methods

### Animals

Adult male (8 to 10 weeks old) C57BL/6 J mice (Jackson Laboratory, Bar Harbor, ME, USA) were housed 6 to 10 per cage under a 12 h dark/light cycle with free access to food and water. Animals weighing 20 to 30 g were used throughout the experiments. The animal protocols were approved by the local Bioethics Commission at the Institute of Pharmacology PAS.

### Drug treatment

Mice were injected i.p. (vol. 10 ml/kg) with ketamine (20 mg/kg), PCP (5 mg/kg) or memantine (15 mg/kg). The animals were killed by decapitation 1, 2, 4 or 8 h after a single injection along with the saline-treated and naive control groups (6 animals per group). The effective doses of the NMDA receptor antagonists were based on the literature, particular attention being paid to their pharmacological effects in C57BL/6 J mice [44–46]. The doses were selected to provide reasonable comparison of drugs’ effects on the molecular level.

### Tissue collection and RNA isolation

Samples containing the hippocampus and striatum were collected. The dissection procedure was performed as previously described [13]. Tissue samples were placed in RNAlater reagent (Qiagen Inc., Valencia, CA, USA) and preserved at −70 °C. Samples were homogenized in 1 ml Trizol reagent (Invitrogen, Carlsbad, CA, USA). RNA was isolated following the manufacturer’s protocol and further purified using the RNeasy Mini Kit (Qiagen Inc.). The total RNA concentration was measured using a ND-1000 Spectrometer (NanoDrop Technologies Inc., Montchanin, DE, USA). The RNA quality was assessed using Agilent Bioanalyzer 2100 (Agilent, Palo Alto, CA, USA).

### Microarray hybridization

A starting amount of 200 ng high-quality total RNA (pooled 1:1 from two animals) was used to generate cDNA and cRNA with the Illumina TotalPrep RNA Amplification Kit (Illumina Inc., San Diego, CA, USA). The resulting cDNA served as a template for in vitro transcription with T7 RNA polymerase and biotin-labeled UTP to generate multiple copies of biotinylated cRNA. Each cRNA sample (1.5 μg) was hybridized overnight to MouseWG-6 BeadChip array (Illumina); subsequently, chips were washed, dried and scanned with the BeadArray Reader (Illumina). Raw microarray data were generated using BeadStudio v3.0 (Illumina). A total of 120 Illumina MouseWG-6 v2 microarrays were used (three independent arrays per group). Samples from 2 mice were pooled per microarray, 3 biological replicates were used per time point and 12 arrays per each drug. Distribution of samples was balanced across array plates and hybridization batches.

### Microarray data analysis

Analysis and quality control of 120 microarrays was performed using BeadArray R package v2.14.1. After background subtraction, the data were normalized using quantile normalization and then log_2_-transformed. The obtained signal was taken as the measure of mRNA abundance derived from the level of gene expression. Statistical analysis of the results was performed using three-way ANOVA (for the drug, time and tissue factors) followed by correction for multiple testing using false discovery rate (percent FDR). The FDR was estimated using the Benjamini and Hochberg method. Furthermore, obtained data were subjected to two-way ANOVA for drug and time as factors for each drug and tissue separately. All statistical analyses were performed in R software version 3.1.1. Gene annotation tool Enrichr was used to identify over-represented ontological groups among the gene expression patterns and to group genes into functional classifications [47]. The visualization of microarray results was performed using dChip software.

### Principal component analysis (PCA) and hierarchical clustering

We used unsupervised hierarchical clustering and principal component analysis (PCA) of the top drug-responsive transcripts to classify the psychotropic drugs. Hierarchical clustering was performed using the measure of Pearson correlation distance and complete distance linkage methods. The gene expression profiles of ketamine (KET), memantine (MEM) and PCP in striatum were combined with previously published profiles of 18 psychotropic drugs from diverse pharmacological and therapeutic classes with saline and naive groups as controls [13]. To reduce batch effects between the experiments, the analyses were performed on log_2_ fold changes over corresponding saline controls. PCA was performed based on expression alterations of the top 50 drug-responsive genes found statistically most significant in two-way ANOVA with drug and time as factors (nominal *p* < 1 × 10^−25^, after Bonferroni correction *p* < 2.3 × 10^−21^). R package FactoMineR version 1.29 was used for the analysis and the PCA was done based on correlation matrix.

### Quantitative PCR

Reverse transcription was performed using Omniscript Reverse Transcriptase (Qiagen Inc.). qPCR reactions were performed using TaqMan® Gene Expression Assays (*Sgk1* - Mm00441387_g1; *Tsc22d3* - Mm00726417_s1; *Plin4* Mm00491061_m1) and isoform-specific TaqMan® probes designed using the Custom TaqMan® Assay Design Tool (Life Technologies, Foster City, CA, USA). The reactions were run on the CFX96 Real-Time system (BioRad). Each template was generated from an individual animal. Samples from two independent experiments were analyzed (*n* = 6 and 8 respectively). Expression of the hypoxanthine-guanine phosphoribosyltransferase 1 (*Hprt1*) transcript was quantified to control for variation in cDNA amounts. The abundance of RNA was calculated as 2^‐ (threshold cycle)^. Data were analyzed using one-way ANOVA followed by Tukey’s HSD multiple comparison test.

### Whole-transcriptome resequencing

High-quality total RNA (1 μg) was ribo-depleted using the RiboMinusTM Eukaryote Kit v2 (Ambion). rRNA-depleted RNA was used as the template for preparation of RNA-seq library generated using the Ion Total RNA-seq Kit v2 system according to the manufacturer’s protocol. For template preparation, we performed emulsion PCR (ePCR) using the Ion OneTouchTM 2 Instrument and the Ion PITM Template OT2 200 Kit v3. Sequencing was performed using an Ion PITM Sequencing 200 Kit v3 and the Ion PITM Chip v2 (Life Technologies). The template-positive ion sphere particles (ISPs) were loaded onto an Ion PITM Chip v2 and sequenced (single end reads >100 bp).

### NGS data analysis

NGS data quality was verified using FastQC. The RNA-seq read alignment was performed using TopHat 2.0.1 and Bowtie 2.1.0. IntersectBed v2.14.2 was used to determine the counts on gene and exon levels. Statistical significance was analyzed using edgeR package v3.10.2. Additionally, the transcript FPKM (Fragments Per Kilobase of transcript per Million fragments mapped) levels were quantified using the Cufflinks package. The analyses were performed using R software v3.0.1. Transcript annotation (biotype and event attributes) and classification was performed using the BioMart interface to the Ensembl gene database [38].

### Ethics approval

The animal protocols were approved by the local Bioethics Commission at the Institute of Pharmacology PAS (no. 910/2012).

### Consent for publication

Not applicable.

### Open access

This article is distributed under the terms of the Creative Commons Attribution 4.0 International License (http://creativecommons.org/licenses/by/4.0/), which permits unrestricted use, distribution, and reproduction in any medium, provided you give appropriate credit to the original author(s) and the source, provide a link to the Creative Commons license, and indicate if changes were made. The Creative Commons Public Domain Dedication waiver (http://creativecommons.org/publicdomain/zero/1.0/) applies to the data made available in this article, unless otherwise stated.

### Availability of data and material

The dataset supporting the conclusions of this article is included within the article and its Additional files 1, 3, 4, 2, 6 and 5. Microarray data were submitted to the NCBI Gene Expression Omnibus (GEO) under accession number [GEO: GSE73800].

## Additional files

Additional file 1: Results from three-way ANOVA for 52 drug-responsive transcripts (FDR for drug factor < 0.01 and absolute fold > 0.5 at least at one time point for any substance) are included. For each transcript a *p*-value and percent FDR for drug, time and tissue factors and all the interactions are presented. (XLSX 19 kb) Additional file 2: The lists of transcript IDs and gene names corresponding to Venn diagrams for the hippocampus and striatum are presented in the first and second sheet respectively. Column names indicate which drugs alter the expression of listed transcripts. In case where only one substance name is listed, the altered expression level is drug-specific (at *p* < 0.0005 from two-way ANOVA for drug and time as factors and absolute fold > 0.3 at least at one of the time points in the analyzed tissue). (XLSX 12 kb) Additional file 3: The results of functional enrichment analysis performed with Enrichr tool for two clusters of genes significantly altered by the selected NMDA antagonists. The table consists of an enriched term, a number of input genes in the pathway (overlap), *p*-value (*p* < 0.05), z-score, combined score (computed as logarithm from *p*-value from the Fisher’s exact test multiplied by the z-score of the deviation from the expected rank) and the overrepresented genes. (XLSX 12 kb) Additional file 4: The lists of probe IDs and gene names obtained in the comparison between tissues based on two-way ANOVA results (at *p* < 0.0005 for the drug factor, absolute fold > 0.3 at least at one of the time points). Transcripts regulated by drug treatment in the hippocampus or striatum specifically and a list of genes altered in both tissues are presented. (XLSX 10 kb) Additional file 5: The pattern of gene expression alterations induced by the selected NMDA receptor antagonists over the time-course (1–8 h after treatment) in the hippocampus and striatum. (PDF 155 kb) Additional file 6: The table summarizing results of gene expression profiling of ketamine effects in the mouse striatum using RNA-seq (*n* = 4). In the first sheet 23 genes regulated by ketamine selected from the microarray profiling are listed with their chromosome location, fold change over control, *p*-value and FDR. The second sheet presents a list of the 15 selected genes with significant differences in expression between ketamine-treated and saline controls (*p* < 0.05) with annotated transcripts abundance and fold change of drug-induced alterations for each regulated transcript. Transcripts were annotated using the Ensembl gene database. For each transcript the analysis on exon level (third sheet) was performed showing exon location on chromosome, fold change, *p*-value, FDR and the number of reads for each exon and sample. The last sheet contains a list of transcript biotypes annotation and classification using the BioMart interface to the Ensembl gene database. (XLSX 81 kb)

## Abbreviations

AFE: alternative first exon
ALE: alternative last exon
BDNF: brain-derived neurotrophic factor
CNE: constitutive exon
GR: glucocorticoid receptor
IR: intron retention
MAPK: mitogen-activated protein kinases
MDD: major depressive disorder
mTOR: mammalian target of rapamycin
NMDA: N-methyl-D-aspartate
PCA: principal component analysis
PCP: phencyclidine
