# Supplementary figures and images for: Molecular profile of dissociative drug ketamine in relation to its rapid antidepressant action
Source: BMC Genomics. 2016 May 17;17:362. doi: 10.1186/s12864-016-2713-3 (PMC4869301; doi:10.1186/s12864-016-2713-3)

## HIPPOCAMPUS

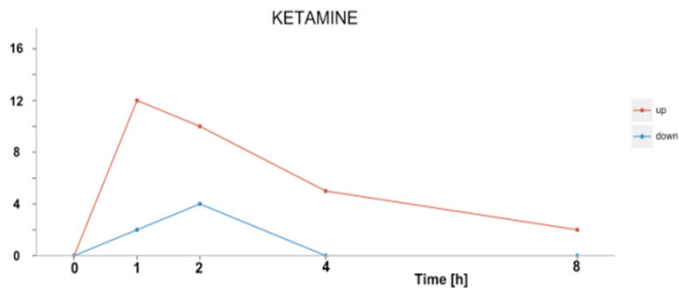

## STRIATUM

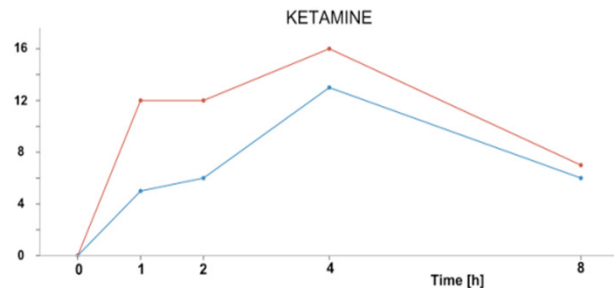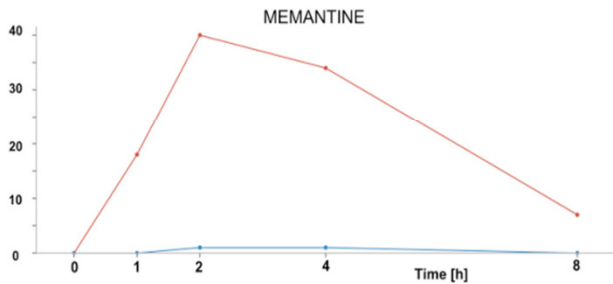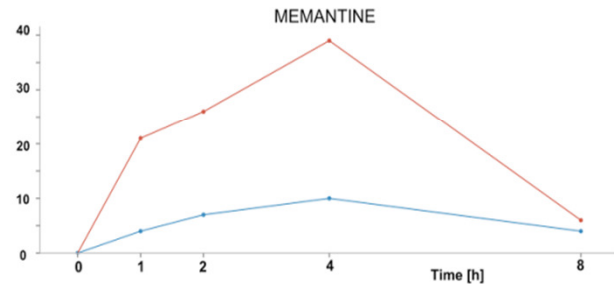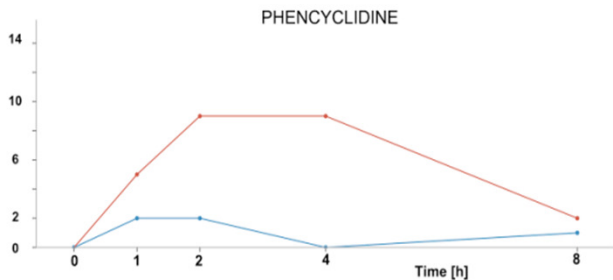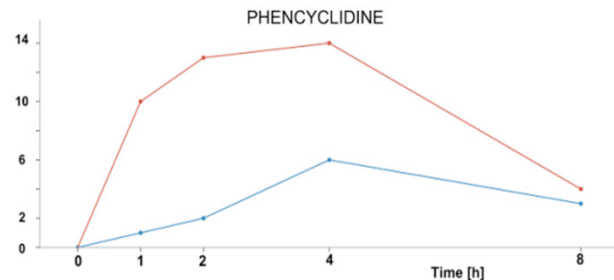

Supplement: Additional file 5: — The pattern of gene expression alterations induced by the selected NMDA receptor antagonists over the time-course (1–8 h after treatment) in the hippocampus and striatum. (PDF 155 kb) [file 12864_2016_2713_MOESM5_ESM.pdf]
